# Atypical cancer risk profile in carriers of Italian founder *BRCA1* variant p.His1673del: Implications for classification and clinical management

**DOI:** 10.1002/cam4.70114

**Published:** 2024-08-28

**Authors:** Giovanni Innella, Cristina Fortuno, Laura Caleca, Bing‐Jian Feng, Courtney Carroll, Michael T. Parsons, Sara Miccoli, Marco Montagna, Daniele Calistri, Laura Cortesi, Barbara Pasini, Siranoush Manoukian, Daniela Giachino, Laura Matricardi, Maria Cristina Foti, Valentina Zampiga, Claudia Piombino, Elena Barbieri, Francesca Vignolo Lutati, Jacopo Azzolini, Rita Danesi, Valentina Arcangeli, Sandrine M. Caputo, Nadia Boutry‐Kryza, Vincent Goussot, Susan Hiraki, Marcy Richardson, Hereditary Breast/Ovarian Cancer IOV network (HBOC IOVnet), Stefania Boni, Milena Gusella, Silvia Tognazzo, Isabella Mammi, Francesco Benedicenti, Licia Turolla, Fiorenza Soli, Simona Ferrari, Paolo Radice, Amanda B. Spurdle, Daniela Turchetti

**Affiliations:** ^1^ Department of Medical and Surgical Sciences (DIMEC) University of Bologna Bologna Italy; ^2^ Medical Genetics Unit Bologna Italy; ^3^ Population Health QIMR Berghofer Medical Research Institute Brisbane Queensland Australia; ^4^ Unit of Predictive Medicine: Molecular Bases of Genetic Risk, Department of Experimental Oncology Fondazione IRCCS Istituto Nazionale dei Tumori Milan Italy; ^5^ University of Utah Salt Lake City Utah USA; ^6^ Immunology and Molecular Oncology Unit Veneto Institute of Oncology IOV—IRCCS Padua Italy; ^7^ Biosciences Laboratory IRCCS Istituto Romagnolo per lo Studio dei Tumori (IRST) “Dino Amadori” Meldola Italy; ^8^ Division of Medical Oncology, Department of Oncology and Hematology University Hospital of Modena Modena Italy; ^9^ Medical Genetics Unit Città della Salute e della Scienza University Hospital Torino Italy; ^10^ Unit of Medical Genetics, Department of Medical Oncology and Hematology Fondazione IRCCS Istituto Nazionale dei Tumori Milan Italy; ^11^ Medical Genetic Unit San Luigi Gonzaga University Hospital Torino Italy; ^12^ Department of Clinical and Biological Sciences University of Turin Torino Italy; ^13^ Romagna Cancer Registry IRCCS Istituto Romagnolo per lo Studio dei Tumori (IRST) “Dino Amadori” Meldola Italy; ^14^ Department of Genetics, Institut Curie, Paris France and Paris Sciences Lettres Research University Paris France; ^15^ Service de génétique Hospices Civils de Lyon Bron France; ^16^ Département de Biologie et Pathologie des Tumeurs Centre de Lutte Contre le Cancer Georges François Leclerc Dijon France; ^17^ GeneDx Gaithersburg Maryland USA; ^18^ Ambry Genetics Aliso Viejo California USA

**Keywords:** *BRCA1*, breast cancer, cancer risk, classification, clinical management, ovarian cancer, penetrance, uterine cancer

## Abstract

**Background:**

*BRCA1*:c.5017_5019del (p.His1673del) is a founder variant relatively frequent in Northern Italy. Despite previous suggestion of pathogenicity, variant classification in public databases is still conflicting, needing additional evidence.

**Methods:**

Maximum likelihood penetrance of breast/ovarian and other cancer types was estimated using full pedigree data from 53 informative Italian families. The effect of the variant on BRCA1‐ABRAXAS1 interaction was assessed using a GFP‐fragment reassembly‐based PPI assay. Results were combined with additional data from multiple sources to classify the variant according to ACMG/AMP classification rules specified for *BRCA1/2*.

**Results:**

Variant‐carriers displayed increased risk for ovarian cancer (HR = 33.0, 95% CI = 7.0–155.0; cumulative risk at age 70 = 27.6%, 95% CI = 12.6–40.0%) but not for breast cancer (HR = 0.7, 95% CI = 0.2–2.2). An increased risk of uterine cancer (HR = 8.0, 95% CI = 1.03–61.6) emerged, warranting further evaluation. Likelihood‐ratio in favor of pathogenicity was 98898642.82 under assumption of standard *BRCA1* breast and ovarian penetrance, and 104240832.84 after excluding breast cancer diagnoses (based on penetrance results). Functional analysis demonstrated that the variant abrogates the BRCA1‐ABRAXAS1 binding, supporting the PS3 code assignment within the ACMG/AMP rule‐based model. Collectively, these findings allowed to classify the variant as pathogenic.

**Conclusion:**

Pathogenicity of *BRCA1*:c.5017_5019del(p.His1673del) has been confirmed; however, breast cancer risk in Italian families is not increased, unlike in families from other countries and in carriers of most *BRCA1* pathogenic variants. The knowledge of atypical risk profiles for this and other variants will pave the way for personalized management based on specific genotype.

## 1 INTRODUCTION

The Italian founder variant c.5017_5019del (p.His1673del) of *BRCA1* (NM_007294.4) is rare world‐wide but is found in a remarkable proportion of families suspected for hereditary breast and ovarian cancer (HBOC) in Italy, particularly in the Emilia‐Romagna region. For this reason, and given its uncertain clinical significance, in 2017 we performed a first study on this variant, suggesting its pathogenicity and observing its association with a predominant ovarian cancer (OC) phenotype.^49^

In that work, the assertion of “pathogenic” was based on a multifactorial likelihood analysis performed according to the method first described by Goldgar et al,^17^ incorporating information on bioinformatic prediction for the amino acid position, co‐segregation in four families, lack of co‐occurrence with another *BRCA1* pathogenic variant in 3827 probands, histopathology of five breast cancer (BC) and nine OC carriers, and tumor loss of heterozygosity (LOH) in four BC and six OC cases. LOH, with likelihood ratios (LR) calculated using the assumed probability distribution described by Chenevix‐ Trench et al.^10^ for BC, provided the highest odds in favor of causality.

Despite this, at July 2023, the *BRCA1* c.5017_5019del (p.His1673del) variant was still reported as “conflicting” in ClinVar (https://www.ncbi.nlm.nih.gov/clinvar/). Further, it had not yet been classified by the Evidence‐based Network for the Interpretation of Germline Mutant Alleles (ENIGMA) consortium (https://enigmaconsortium.org), considered the main source for interpretations of *BRCA1* and *BRCA2* variants according to Italian Scientific Societies consensus.^37^ As such, many Italian laboratories were reporting *BRCA1* c.5017_5019del (p.His1673del) as a variant of uncertain significance (VUS), leading to differences in management recommendations between and (potentially) within families. Moreover, the original general observation that carriers within the families were more likely to present with OC than BC^49^ raised the possibility that this variant may exhibit a penetrance profile different from the average *BRCA1* truncating variant, potentially justifying nuanced clinical management recommendations.

For these reasons, we decided to undertake formal penetrance analysis to estimate cancer risks associated with the *BRCA1* c.5017_5019del (p.His1673del) variant, to experimentally test the effect of the variant on BRCA1 protein function, and to re‐assess its clinical significance using updated classification protocols.

## 2 MATERIALS AND METHODS

### 2.1 Ascertainment of families

Families were ascertained on the basis of presentation of at least one individual with personal or family history suggestive of HBOC, with the index case identified to be a carrier of the c.5017_5019del (p.His1673del) variant. The dataset included updated pedigrees from our previous study,^49^ and new pedigrees. The data were collected from centers located in different Italian hospitals (Bologna, Modena, Meldola, Milano, Padova, Torino, and Orbassano) with long‐standing experience in cancer genetics, and adopting procedures aimed at increasing accuracy of family history collection, with verification of cancer diagnoses in all tested cases and, whenever possible, in affected relatives. Pedigrees and individual data were collected from a total of 72 families, 53 of which had at least one additional person genotyped other than the index case, and were thus informative for co‐segregation analysis and for estimating cancer risks. Additional details are reported in Supplementary—Data S1.

### 2.2 Penetrance analysis

Penetrance analysis was undertaken for BC, OC, prostate cancer, pancreatic cancer, colorectal cancer and uterine cancer, selected based on previous association with *BRCA1* (or *BRCA2*) pathogenic variant status, or the number of affected individuals with that tumor in the dataset. Risks were estimated using modified segregation analysis with the MENDEL package of programs.^26^ The risks for each cancer under study were estimated independently, censoring each affected individual at their age at the first occurrence of the specific tumor. For BC and OC, the risks were also calculated considering both tumors simultaneously. To account for ascertainment bias, the likelihood of the pedigree phenotypes and c.5017_5019del (p.His1673del) genotypes was calculated conditional on the pedigree phenotypes and the variant genotype of the index case.

For BC and OC, we estimated the hazard ratio (HR) associated with the development of the tumor under the assumption that the HR across age‐groups was constant (with both the simultaneous and independent model), and assuming that the HR was different across the age‐groups <50 and ≥50 years (only with the independent model); this cut‐point was selected as optimal after trying others because it featured the tightest CIs and the highest log‐likelihood values. For other cancer types, because of lower number of cancer diagnoses, we calculated risks only assuming a constant HR across ages. Baseline population incidence rates were set to be those for the Italian population 2003–2007 (Cancer Incidence in Five Continents Reports).^15^ In the main analysis for OC, population incidence data related to all ovarian neoplasms were used, but a secondary analysis considering data related to only epithelial tumors and tubaric cancers was also performed (Supplementary—Data S1).

From the resulting estimates of relative risk for each tumor type, age‐specific cumulative risk estimates were calculated based on the cumulative incidence $\Lambda \left(t\right):F\left(t\right)=1-\exp\left(-\Lambda \left(t\right)\right)$, and the corresponding CIs were calculated using a parametric bootstrap.

### 2.3 Functional assay

We assessed if the *BRCA1* c.5017_5019delCAC (p.His1673del) variant affects the interaction of the BRCA1‐BRCT domains with the binding protein ABRAXAS1, as performed in previous studies (rational and details in Supplementary—Data S1),^6^, ^7^ using the following methods:

#### 2.3.1 Plasmids and site‐directed mutagenesis

The pET11a‐NfrGFP‐BRCA1 and pMRBAD‐ABRAXAS1‐Cfr Green Fluorescent Protein (GFP) were described in Caleca and Radice (2023).^7^ The selected *BRCA1* variants were introduced by PCR‐mediated directed mutagenesis of pET11a‐NfrGFP‐BRCA1 using the QuikChange II site‐directed mutagenesis kit (Agilent Technologies, Santa Clara, CA, USA) according to the manufacturer's instructions. The presence of variants in recombinant clones was verified by DNA sequencing (Eurofins Genomics, Ebersberg, Germany).

#### 2.3.2 Green Fluorescent Protein (GFP)‐fragment reassembly screening

ArcticExpress (DE3) *E. coli* competent cells (Agilent, Santa Clara, CA, USA) were co‐transformed by heat‐shock method with the compatible pairs of plasmids (pMRBAD‐ABRAXAS1‐CfrGFP and pET11a‐NfrGFP‐BRCA1, both as wild‐type and mutant forms) and screened for the occurrence of the GFP‐fragment reassembly, as previously described.^6^ Fluorescence images were captured after excitation with long‐wave (365 nm) UV light using Azure 600 Imaging System (Dublin, CA, United States) as specified by the manufacturer. All pictures were taken with the same setting of instrument.

#### 2.3.3 *E*.*coli* cell extracts preparation

ArcticExpress (DE3) *E*. *coli* cell extracts were derived as described in Caleca and Radice (2023).^7^ The protein concentration was determined by the Bradford method using the Bio‐Rad protein assay kit (Bio‐Rad Laboratories, Hercules, CA, USA) according to manufacturer's instructions. Equal amounts of protein (20 μg) were subjected to 4%–20% precast gradient polyacrylamide gel (Bio‐Rad Laboratories, Hercules, CA, USA) and visualized by Western blotting using a polyclonal anti‐GFP antibody (#A1026‐2, Thermo Fisher Scientific, Eugene, Oregon, USA) as a primary antibody and goat anti‐chicken IgY(H + L) (#A16054, Invitrogen Rockford, IL,USA) as a secondary antibody.

### 2.4 Variant classification

We revisited classification using the American‐College‐of‐Medical‐Genetics‐and‐Genomics and Association‐for‐Molecular‐Pathology (ACMG/AMP) guidelines^36^ now in common usage, and the standard adopted for ClinGen variant curation expert panels (VCEPs). All data previously included in our multifactorial likelihood analysis were assessed for overlap, and consistency/applicability of weights applied for different evidence types. In particular, we reassessed the value of breast and ovarian tumor LOH for predicting variant pathogenicity, by reviewing publicly available information on the rate of *BRCA1* LOH in sporadic OC, and published information on breast and ovarian tumor *BRCA1* LOH as a predictor of variant pathogenicity^10^, ^38^, ^46^; from this exercise, we decided to discard use of LOH for both BC and OC in revised multifactorial analysis (details in Supplementary Data S1, including Tables S1 and S2). Regarding other factors, we performed a co‐segregation analysis of our updated dataset using the online tool Co‐segregation Online v3 (https://fengbj‐laboratory.org/cool3/analysis.html),^3^ under two different assumptions: (i) a standard analysis considering all tumors occurring in the individuals present in the dataset; (ii) an analysis considering individuals affected by BC as unaffected at age at BC diagnosis, as justified by penetrance analysis results (see “Results” section). In addition, the analysis was also performed on four French families from French UnicancerGeneticsGroup (UGG) using updated information for pedigrees as originally published,^8^ for one family from GeneDX and for five families from AmbryGenetics. Tumor pathology LRs were calculated as described in Spurdle et al. for BC^45^ and in O'Mahony et al. for OC.^32^ These data were combined with other information from the literature and from unpublished data collated by ENIGMA consortium members to obtain a total LR based on multiple independent clinical data types, either assuming standard *BRCA1* cancer penetrance, or by censoring all results derived or potentially derived from BC cases.

The combined LR based on clinical data was integrated with other information to determine the clinical significance of c.5017_5019del (p.His1673del) through the application of *BRCA1/2* specified ACMG/AMP classification rules, developed by the ENIGMA VCEP (Classification Criteria V1.0 2023‐04‐27—https://clinicalgenome.org/affiliation/50087/), as recently performed for all the VUSs detected in our center.^19^ These classification criteria allow for use of LR evidence derived from previously reported multifactorial likelihood analyses, and for application of LR‐based weights for evidence types not formally recognized in the baseline ACMG/AMP criteria.^36^

## 3 RESULTS

### 3.1 Italian cohort description and cancer features

The cohort included 791 individuals from 72 Italian families identified to carry the c.5017_5019del(p.His1673del) variant. A summary of cancer history, age distribution, and carrier status is shown in Table S3.

A total of 243 individuals were genotyped, with 180 confirmed to be carriers of the variant: 150 females and 30 males. No other *BRCA1/2* variants were detected in index cases. Among women, there were 86 cases of BC (39 carriers, 10 non‐carriers, 37 untested), 90 cases of OC (65 carriers, 25 untested), two cases of pancreatic cancer (both untested), nine cases of colorectal cancer (2 carriers, 1 non‐carrier, 6 untested) and nine cases of uterine cancer (4 carriers, 5 untested). Among men, there were three cases of prostate cancer (1 carrier, 2 untested), four cases of colorectal cancer (1 carrier, 3 untested), and no cases of BC or pancreatic cancer.

Main clinical‐pathological features of BC and OC in carriers are summarized in Table S4. BCs were diagnosed at an average age of 50 years and most frequently at an early stage (stage I 50.0%, stage II 42.3%), and most tumors were ductal type (83.9%), high‐grade (55.6%) and hormone‐responsive rather than triple‐negative (65.5% vs. 35.7%). OCs were diagnosed at an average age of 56 years and most frequently at an advanced stage (stage III 57.5%, stage IV 7.5%), and most tumors were of high‐grade (97.9%) and serous histologic type (95.4%).

### 3.2 Risk estimates

Table 1 shows HR and cumulative risk estimates for different cancers in carriers of c.5017_5019del(p.His1673del) calculated assuming constant risk across age groups, and for BC/OC also modeling risks for <50 and ≥50 years age groups.

**TABLE 1:** HRs and cumulative risks estimates for Italian carriers of c.5017_5019del (p.His1673del) variant assuming constant risk across age groups, and modeling risks for <50 and ≥50 years age groups for breast and ovarian cancer.

Tumor	Sex	Model^a^	HR (95% CI)	% Cumulative risk (95% CI)	
				Age 50 years	Age 70 years
BC	F	Constant risk (simultaneous analysis)	0.7 (0.2–2.2)	2.5 (1.1–3.9)	6.7 (4.9–9.3)
		Constant risk (independent analysis)	0.8 (0.3–2.1)	2.6 (1.2–4.0)	6.9 (4.3–9.4)
		Age‐dependent risk (independent analysis)	<50 years: 1.3 (0.4–4.5)	4.4 (1.2–11.9)	7.8 (2.3–19.0)
			≥50 years: 0.4 (0.1–2.0)		
OC	F	Constant risk (simultaneous analysis)	33.0 (7.0–155.0)	9.9 (2.5–16.7)	27.6 (12.6–40.0)
		Constant risk (independent analysis)	45.5 (8.8–236.1)	14.1 (3.0–24.0)	38.2 (16.7–54.2)
		Age‐dependent risk (independent analysis)	<50 years: 31.1 (3.3–292.4)	19.8 (3.2–62.8)	51.9 (11.1–99.4)
			≥50 years: 71.6 (7.4–692.6)		
ProC	M	Constant risk (independent analysis)	1.5 (0.1–17.7)	1.9 (0.0–4.2)	12.1 (0.0–25.9)
CRC	M	Constant risk (independent analysis)	3.4 (0.5–24.4)	1.9 (1.7–2.2)	15.8 (0.8–28.6)
	F	Constant risk (independent analysis)	4.3 (0.6–30.6)	2.2 (0.0–4.7)	12.7 (1.7–22.5)
UC	F	Constant risk (independent analysis)	8.0 (1.0–61.6)	2.8 (0.0–6.0)	12.3 (2.0–21.5)

^a^ In the independent analysis, only individuals with the tumor under examination are considered affected, while in the simultaneous analysis, both individuals with BC and those with OC are considered affected.

Abbreviations: BC, Breast Cancer; CI, Confidence Interval; CRC, ColoRectal Cancer; HR, Hazard Ratio; OC, Ovarian Cancer; ProC, Prostate Cancer; UC, Uterine Cancer.

There was no evidence for significantly increased risk of BC relative to the general population from the constant model or age‐dependent analyses. In contrast, acknowledging wide and overlapping CIs for the different models, the HR for OC was high for the constant model (simultaneous analysis: HR 33.0, 95% CI 7.0–155.0; independent analysis: HR 45.5, 95% CI 8.8–236.1) and the age‐dependent model (<50 years: HR 31.1, 95% CI 3.3–292.4; ≥50 years: HR 71.6, 95% CI 7.4–692.8). Based on the simultaneous analysis performed with the constant model, the resulting cumulative risks at 70 years of age were calculated as 6.7% (95% CI 4.9%–9.3%) for female BC, and 27.6% (95% CI 12.6%–40.0%) for OC. Risk estimates for BC and OC under alternative assumptions yielded consistent results (Tables S5 and S6).

Of the other cancer types analyzed, constant model analysis also estimated statistically significant HR >1.0 associated with the variant for uterine cancer (HR 8.0, 95% CI 1.03–61.7), translating to a cumulative risk at 70 years of age of 12.3% (95% CI 2.0%–21.5%).

**FIGURE 1:**
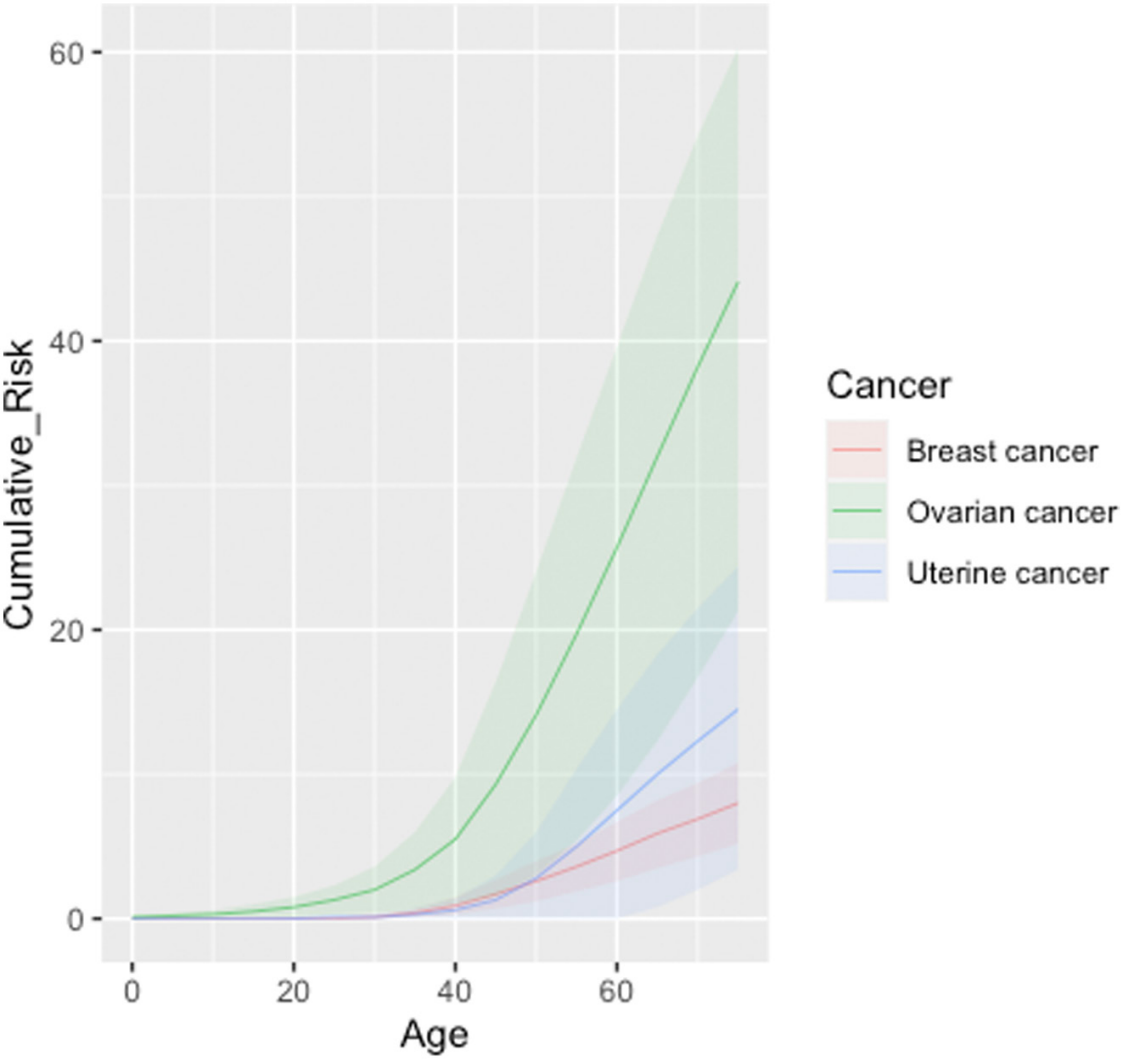
Cumulative risk (%) estimates for breast, ovarian and uterine cancer in female Italian carriers of c.5017_5019del (p.His1673del) variant. Estimates were calculated assuming a constant HR across age groups, with simultaneous analysis for BC and OC, and with independent analysis for UC.

Cumulative risks for breast, ovarian and uterine cancer are represented in Figure 1.

### 3.3 Functional results

**FIGURE 2:**
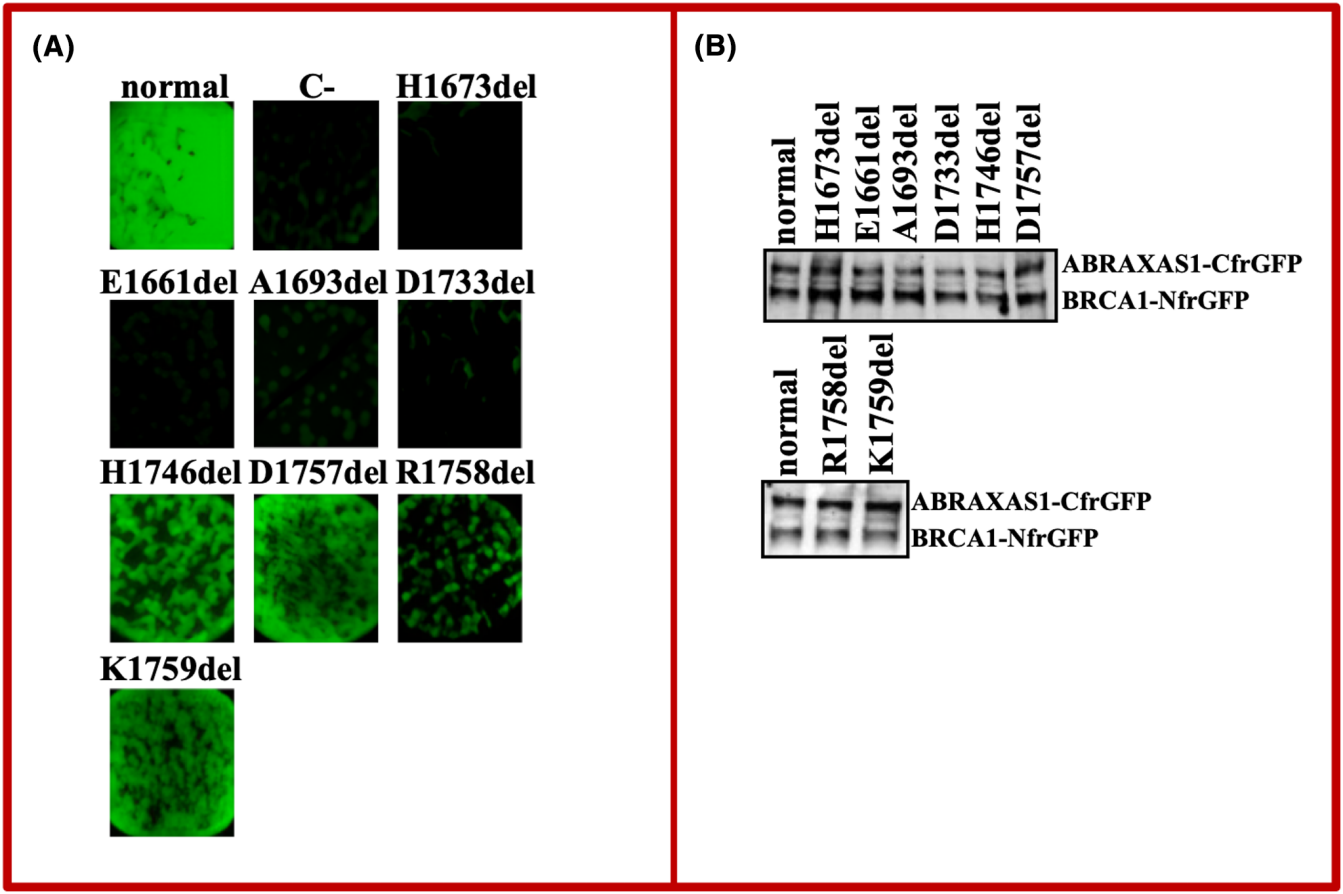
Detection of the BRCA1‐ABRAXAS1 interaction. (A) In vitro GFP‐reassembly assays. Fluorescence was recovered, under long‐wave UV light (365 nm), after 24 h of growth at 37°C followed by 3 days of incubation at room temperature. Arctic Express (D3) *E*.*coli* bacterial cells were co‐transformed twice with each compatible pair of plasmids. C‐: Arctic Express (D3) *E.coli* bacterial cells co‐expressing non‐cognate BRCA1 and Leucine zipper fusion pepetides as negative control. (B) Analysis of expression of BRCA1‐NfrGFP and ABRAXAS1‐CfrGFP wild‐type and mutant forms. Cell extracts from the co‐transformed ArcticExpress (DE3) *E*. *coli* cells were subjected to SDS‐PAGE and visualized by Western blotting using a polyclonal anti‐GFP antibody.

A bright fluorescence, as a result of protein–protein interaction (PPI), was observed in bacterial cells co‐expressing ABRAXAS1 together with normal BRCA1 protein, whereas no fluorescence was observed in bacterial cells co‐expressing ABRAXAS1 together with BRCA1 carrying the p.His1673del, indicating that the variant affects PPI (Figure 2). This result is in keeping with that reported in a previous study that assessed the p.His1673del variant as functionally impaired, using an extensively validated transcriptional activity assay.^31^ To verify the robustness of our assay in discriminating between functionally proficient and functionally inactivating small in‐frame indels, we tested seven additional *BRCA1*‐BRCT variants of this type that were examined in the study by Nepomuceno et al.^31^ (Figure 2A), and the results of the two assays were totally consistent (Table 2).

**TABLE 2:** Comparison between results from Nepomuceno et al (2022)^31^ and those of this study.

Nucleotide variant^a^	HGVS protein^b^	ACMG code^c^	ABRAXAS1 interaction^d^
c.5017_5019del	p.(His1673del)	PS3	No
c.4981_4983del	p.(Glu1661del)	PS3	No
c.5078_5080del	p.(Ala1693del)	PS3	No
c.5197_5199del	p.(Asp1733del)	PS3	No
c.5238_5240del	p.(His1746del)	BS3	Yes
c.5269_5271del	p.(Asp1757del)	BS3	Yes
c.5272_5274del	p.(Arg1758del)	BS3	Yes
c.5275_5277del	p.(Lys1759del)	BS3	Yes

^a^ NM_007294.3.

^b^ NP_009225.1.

^c^ Based on functional assessment by TA assay.^31^

^d^ This study.

Specifically, four variants that were previously observed not to significantly alter transcriptional activity compared to normal control (p.His1746del, p.Asp1757del, p.Arg1758del, p.Lys1759del) did not interfere with ABRAXAS1 binding, whereas the three variants showing strongly reduced transcriptional activity (p.Glu1661del, p.Ala1693del, p.Asp1733del) prevented PPI. Similar levels of protein expression from all mutant constructs were detected, demonstrating that when in the in vitro GFP‐reassembly screening a loss of fluorescence was observed, this was attributable to the lack of binding between the proteins and not to poor expression of the mutants (Figure 2B). The locations of the tested variants in the BRCA1 protein model is shown in Figure S1.

### 3.4 Variant classification following ACMG/AMP guidelines

Results of co‐segregation analysis and pathology LR analysis of our expanded Italian dataset are summarized in Tables S7–S9. Standard co‐segregation analysis in our cohort provided “strong” evidence toward pathogenicity of the c.5017_5019del(p.His1673del) variant (LR 114.61). Censoring BC cases as justified by penetrance results (which showed no associated risk for BC), the LR increased to 3646.25, representing “very strong” evidence. Tumor pathology, based on 31 BCs and 2 high‐grade endometrioid OCs (a type that unlike high‐grade serous cancer is considered informative by O'Mahony et al (2023)^32^), provided contrasting results: evidence against pathogenicity for BC (LR 0.0004), and in favor for OC (LR 8.88).

The results on the expanded Italian dataset were integrated with those of other unrelated datasets available in literature and/or provided by collegues from outside Italy in order to obtain a combined LR based on multiple independent clinical data. For the additional datasets, we calculated the LR both in a standard way, and also excluding data relating to BC cases as justified by penetrance results from Italian families: the total LR was 98898642.82 using the standard approach, and 104240832.84 excluding BC‐related information, both scenarios representing very strong evidence toward pathogenicity of the variant. These results, shown in Table 3, were used together with other types of information for variant classification, as detailed in Table 4.

**TABLE 3:** Calculation of multifactorial likelihood ratio derived from independent clinical data assuming standard penetrance (Main analysis) and revised penetrance^a^ (Secondary analysis).

Feature	Source	Specifications	LR	
			Main analysis	Secondary analysis
Co‐segregation	COVAR (Caputo et al 2021, revised and updated)^8^	4 families	4526.46	4.57
	GC‐HBOC^34^	3 families	1.59	1.59
	GeneDx (unpublished)	1 family	1.46	1.46
	Ambry Genetics (unpublished)	5 families	1775.08	304.41
	This dataset (Zuntini et al 2017, revised and updated)^49^	53 families	114.61	3646.25
	Total Co‐segregation	66 families	2132877421.28	11738303.77
Pathology	COVAR (Caputo et al 2021, revised and updated)^8^	5 BC	0.41	n/a
	GC‐HBOC^34^	2 BC	1.40	n/a
	Bang et al, 2022^b^.^2^	10 BC	13.11	n/a
	Ambry Genetics (unpublished)	1 BC	3.73	n/a
	This dataset (Zuntini et al 2017, revised and updated)^49^	31 BC (LR 0.0004) and 2 OC (LR 8.88)	0.003	8.88
	Total Pathology	49 BC and 2 OC	0.09	8.88
Personal & Family history	COVAR (Caputo et al 2021)^8^	6 families	0.49	n/a
	Total Personal and Family History	6 families	0.49	n/a
Total			98898642.82	104240832.84

Abbreviations: BC, Breast Cancer; LR, Likelihood ratio; n/a, Not Applicable; OC, Ovarian Cancer.

^a^ As justified by penetrance findings for Italian families, we excluded data related to BC cases in Secondary analysis (for segregation analysis, individuals with BC were censored as unaffected at age of BC).

^b^ The variant has been described in the Korean population by several papers,^18^, ^23^, ^33^, ^42^ to avoid potential data overlap we chose to include data only from Bang et al (2022)^2^ because it is the most recent publication and the one with the most information.

**TABLE 4:** Assignment of ACMG/AMP classification criteria toward pathogenicity for c.5017_5019del (p.His1673del) variant^a^.

Criterion	Assignment		Weight
PVS1	n/a		
PS1	n/a		
PS3	A validated functional assay performed by Nepomuceno et al (2022)^31^ showed that the c.5017_5019del (p.His1673del) variant is associated with abrogation of transcriptional activity and a marked reduction of BRCA1 protein levels, results supported by the functional study performed in present study (Figure 2 and Table 2). This evidence strongly supports the pathogenicity of the variant.	✓	Strong
PS4	No relevant data.	✗	/
PM2	Not seen in GnomAD, but PM2_Supporting is not applied for indels, due to poor recall for this variant type^13^	✗	/
PM3	Observed “in trans” with a *BRCA1* pathogenic variant (c.1116G > A;p.Trp372Ter) in a patient with a phenotype consistent with Fanconi Anemia (small for gestational age, laryngotracheomalacia, hypoglycemia, respiratory distress, monolateral congenital cataract, postnatal severe growth retardation, dysmorphic features, malignant brain tumor at 13 months).^4^ Mitomycin C‐induced chromosomal breakage test performed in patient's peripheral blood lymphocytes showed greatly reduced proliferation but no evidence of increased chromosomal breakage, which would theoretically be required to meet the criterion PM3. However, since it is recommended that cases strongly suspected for Fanconi Anemia with no evidence of chromosomal breakage on blood should repeat the test on skin fibroblasts (https://www.fanconi.org/explore/clinical‐care‐guidelines),^1^, ^9^ we decided to apply the criterion PM3 with a “supporting” weight based on clinical evidence.	✓	Supporting
PM5_PTC	n/a		
PP1	These data, summarized in Table 2 and Table S7, are integrated in the PP4 criterion with other clinical data, as per ClinGen *BRCA1/2* VCEP specifications.	✗	/
PP3	Missense alteration is located inside a (potentially) clinically important functional domain of the *BRCA1* gene (C‐terminal (BRCT) domain), but BayesDel score of 0.02 suggests no impact on protein function. Effect on splicing calculated with SpliceAI provided a non‐informative value (0.13), so bioinformatic criteria could not be applied.	✗	/
PP4	Multifactorial likelihood ratio derived from independent clinical data (tumor pathology features, segregation and personal and family history data, details in Table 2), provided a combined LR toward pathogenicity of 98898642.82 assuming standard penetrance, and combined LR of 104240832.84 assuming association with ovarian cancer only (as justified by penetrance results for Italian families). In both cases, the criterion PP4 with a “very strong” weight could be applied.	✓	Very Strong

Abbreviationsa: n/a, criterion not applicable; ✓, criterion met; ✗, criterion not met.

^a^ The variant did not meet any criteria toward benignity.

Overall, the variant satisfied the “PS3”, “PM3” and “PP4” criteria, with a “strong”, “supporting” and “very strong” weight respectively (equivalent to 13 points), reaching classification as “pathogenic” according to the ACMG/AMP classification rules specified for *BRCA1/2*.^19^

## 4 DISCUSSION

Following our previous publication on the Italian founder variant c.5017_5019del (p.His1673del),^49^ the clinical centers participating in the study started to manage it as likely pathogenic, offering carriers clinical management as for carriers of pathogenic *BRCA1/2* variants, and performing cascade testing for family members. However, the variant has still been reported as VUS by some diagnostic laboratories within and outside Italy, based on ClinVar reports. In the Italian context there was interest to clarify if variant‐associated risks were similar to those conferred by classic *BRCA1* variants, to better inform appropriate clinical management options.

Penetrance analysis estimated for carriers a high risk for OC with all the different model used (HR 33.0 and cumulative risk at 70 years of 27.6% with the simultaneous age‐constant analysis), comparable to that associated with truncating *BRCA1* variants^25^, ^27^; this is also supported by the fact that the features of OC diagnosed in carriers of the variant are those typical of *BRCA*‐associated tumors (Table S4).^20^ In contrast, results indicated no evidence of an increased risk for BC for our Italian cohort (HR 0.7 and cumulative risk at 70 years of 6.7% with the simultaneous age‐constant analysis), a finding supported by strong evidence against pathogenicity based on breast tumor features in these families (LR 0.00038). These data refer to female cases, as the analysis carried out in male patients did not show a significant increase in risk for the tumors analyzed, that is, BC (0 cases), pancreatic cancer (0 cases), prostate cancer (three cases, of which one carrier of the variant and two untested), and colorectal cancer (four cases, of which one carrier of the variant and three untested).

Using updated data from the Italian cohort, plus other previously published and newly acquired unpublished data, the variant was classified as pathogenic following gene‐specific ACMG/AMP guidelines for different types of evidence. First, its impact on protein function was demonstrated in vitro by a recent functional study^31^ and by the GFP‐fragment reassembly‐based PPI assay performed in this study, which provided experimental evidence that it abrogates the BRCA1‐ABRAXAS1 binding supporting the PS3 code assignment within the ACMG/AMP rule‐based model.^5^, ^28^ Then, its co‐occurrence “in trans” with an established pathogenic variant in an individual with a clinical phenotype consistent with Fanconi Anemia (Table 4) confirmed in vivo a functional impairment associated with the variant.^4^ Finally, the pathogenicity was supported by the results of independent clinical data analysis performed following a standard *BRCA1* penetrance model and also after excluding data relating to BC diagnoses, as justified by the penetrance results (Table 3). Interestingly, secondary analysis considering individuals with BC as unaffected resulted in increased co‐segregation LR for Italian families (114.61 to 3646.25) but decreased co‐segregation LR for four large French families (4526.45 to 4.57) and for five families from Ambry Genetics (1775.08 to 304.41). In addition, there was a marked difference in the combined LR based on BC features for Italian families (LR 0.0004) compared to that from carriers in families outside of Italy (LR 18.65). These observations suggest need to conduct penetrance analysis of families from outside Italy, and to consider possible modifying factors that may contribute to heterogeneous presentation. Given that Italian families have previously been shown to occur on a single haplotype,^49^ the possibility of cis‐regulatory effects should not be discounted. Regardless, our findings demonstrate how the unusual presentation of specific variants can complicate formal classification using criteria based on “standard” presentations for a given gene.

It is known that different variants of the same tumor predisposition gene can confer different levels of risk and, regarding *BRCA1/2* genes, there is robust evidence that the *BRCA1* p.Arg1699Gln variant is associated with reduced penetrance of BC and OC, compared to the average (largely truncating) *BRCA1* variants.^29^, ^47^ More recently, it has also been reported that BC risk after age 50 years (but not at younger ages) was lower for women with *BRCA1* pathogenic missense variants as a group, compared to those with protein termination codon variants,^27^ but there was no evidence for difference in OC risk between the variant types. The c.5017_5019del (p.His1673del) variant—at least in the Italian context—appears to represent another presentation, with BC risk not different to that of the general population, but OC risk at least comparable to that conferred by truncating *BRCA1* variants. It is notable that six women in our study presented with OC prior to BC. At present, we can only speculate as to the biological explanation for differing presentation: although the c.5017_5019del (p.His1673del) variant does not fall into the previously described OC cluster region,^35^ it is possible that a deletion variant has different properties to that of a missense change at a given residue, for example, if the protein backbone is impacted by a deletion variant (but not a missense change). Moreover, its occurrence “in trans” with a variant predicted to encode a truncated non‐functional BRCA1 protein (c.1116G > A;p.Trp372*) in a patient with a phenotype suggestive for Fanconi Anemia^4^ further supports its peculiar biological effect and differences in penetrance profile, since patients with biallelic *BRCA1* variants have been reported to carry at least one variant with reduced penetrance or potential rescue mechanisms resulting in some level of retained protein function.^14^, ^22^, ^39^

It was not possible to compare the risk of other tumors typically associated with pathogenic *BRCA1/2* variants (particularly pancreatic and prostate cancers) associated with the c.5017_5019del (p.His1673del) variant with that conferred by other *BRCA1/2* variants because of the limited number of cases with such tumors in this series. However, penetrance analysis did not show a significantly increased risk for these tumor types.

Interestingly, penetrance analysis also highlighted a significant increase of uterine cancer risk in carriers of the c.5017_5019del (p.His1673del) variant (HR: 8.0, 95% CI = 1.03–61.6; cumulative risk at 70 years: 12.3%, 95% CI = 2.0%–21.5%) compared to the general population (about 3.1%^40^). This finding is consinstent with several studies reporting a significant increase of the risk for endometrial cancer in carriers of *BRCA1/2* pathogenic variants, especially for *BRCA1* gene (but not for specific variants), with some suggesting an association specifically for the serous histotype.^16^, ^21^, ^24^, ^30^, ^41^, ^44^ Although this evidence is not yet clearly established, recommendations exist to consider risk‐reducing hysterectomy in carriers of *BRCA1* pathogenic variants.^12^, ^43^ However, the estimates calculated here are based on only 9 cases, which were actually considered uterine (and not ovarian) tumors for the clinical history of the affected patient, but of which only two were histologically verified. Therefore, to provide more definitive conclusions in this regard, it would be important to study a larger number of cases and consider information on uterine tumor subtypes.

To date, in Italy, female carriers of *BRCA1/2* pathogenic variants are clinically managed according to regional protocols, which follow recommendations provided by Italian scientific societies (https://www.aiom.it) and include intensive breast surveillance (MRI‐based) starting at young age (25/30 yaers) and optional risk‐reducing mastectomy. The findings from this work have important implications for family handling (all centres should consider the variant as pathogenic for cancer risk), and personalized clinical management for carriers of the variant c.5017_5019del (p.His1673del). The risk estimates from the present study confirm the appropriateness of current OC risk management, while questioning the indication for prophylactic breat surgery or particularly intensive breast surveillance. At least for Italian families, the present results suggest a BC risk management based on family history through a case by case evaluation and pave the way for future genotype‐based management. Furthermore, the evidence of an increased risk for uterine cancer would suggest continuing to remain vigilant on gynecological symptoms even after eventual prophylactic oophorectomy. However, caution must be exercised in applying specific clinical management to carriers of this variant, and case‐specific assessments must be carried out, since there is a large variability between Italian and non‐Italian families, but also between different Italian families.

Finally, the origin of most of our families further support the hypothesis that the variant has arisen in a common ancestor presumably living in Northern Italy. However, this variant has also been reported in other populations,^2^, ^8^, ^18^, ^34^ suggesting that it may fall into a mutational hot‐spot; haplotype analysis of carriers from different cohorts would be necessary to further investigate this hypothesis. Anyway, the fact that this variant is also present in other populations highlights the importance of findings from this study, which indicate that the variant displays evidence toward pathogenicity using multiple evidence types, and can be classified as pathogenic following the ACMG/AMP system.

For the component of work focused on the Italian cohort, the main limitations of this study are: (1) the study design as a retrospective family‐based study, where family history in some instances could not be verified (however, 51.2% of BCs, 60.0% of OCs and 22.2% of uterine cancers were verified through clinical records); (2) the lack of age at diagnosis for some cancers, even if in a limited percentage (3.4% of BCs, 2.2% of OCs, 15.4% of colorectal cancers and 11.1% of uterine cancers), which had to be imputed; (3) the lack of tumor histopathological data for some carriers (21.1% of BC cases and 33.9% of OC cases), with consequent reduction of the sample on which the pathology LR was calculated, and for cancers generally considered to be outside the spectrum of *BRCA1*‐related disease, particularly relevant for uterine cancer analyses. Another concern is the limitation in extent and detail of information from families from outside Italy. In particular, due to restrictions in data sharing, it was not possible to conduct formal penetrance analysis for non‐Italian families; however, non‐Italian collaborators conducted co‐segregation analysis with and without consideration of BC as a cancer of interest as a means to replicate the Italian findings of increased OC but not BC risk for this variant. For these reasons, and given that results in French families and families from Ambry Genetics were contrasting with those obtained for the Italian cohort, it will be important to collect additional data and unrelated families especially from outside Italy, and to consider the possibility of haplotype as a modifier of BC risk, in order to provide more guidance on clinical management of carrier families.

Further, since it is known that carriers of *BRCA1/2* pathogenic variants who develop BC have a different prognosis and clinical outcome compared to non‐carriers,^11^, ^48^ another aspect that would be interesting to evaluate in the future is whether the c.5017_5019del (p.His1673del) variant, in addition to being associated with a different risk of breast cancer compared to other *BRCA1* variants, is also associated with a different prognosis. For this reason, it will be important to prospectively collect clinical data from BC patients carrying this variant.

## 5 CONCLUSION

This work, performed with combined methodological approaches on a larger cohort of families compared to our previous study on the variant c.5017_5019del (p.His1673del), has provided estimates of variant‐associated cancer risks within Italian families, and formal variant classification following the ACMG/AMP guidelines considering clinical data from Italian and other families. These findings indicate the importance of considering variant‐specific differences in cancer risk presentation, and implications for variant classification and informed practice of personalized medicine.

## AUTHOR CONTRIBUTIONS

**Giovanni Innella:** Conceptualization (equal); data curation (equal); formal analysis (equal); investigation (equal); writing – original draft (lead); writing – review and editing (equal). **Cristina Fortuno:** Data curation (equal); investigation (equal); methodology (equal). **Laura Caleca:** Data curation (equal); investigation (equal); methodology (equal). **Bing‐Jian Feng:** Data curation (equal); investigation (equal); methodology (equal). **Courtney Carroll:** Data curation (equal); investigation (equal); methodology (equal). **Michael T. Parsons:** Data curation (equal); investigation (equal); methodology (equal). **Sara Miccoli:** Data curation (equal); investigation (equal). **Marco Montagna:** Data curation (equal); investigation (equal). **Daniele Calistri:** Data curation (equal); investigation (equal). **Laura Cortesi:** Data curation (equal); investigation (equal). **Barbara Pasini:** Data curation (equal); investigation (equal). **Siranoush Manoukian:** Data curation (equal); investigation (equal). **Daniela Giachino:** Data curation (equal); investigation (equal). **Laura Matricardi:** Data curation (equal); investigation (equal). **Maria Cristina Foti:** Data curation (equal); investigation (equal). **Valentina Zampiga:** Data curation (equal); investigation (equal). **Claudia Piombino:** Data curation (equal); investigation (equal). **Elena Barbieri:** Data curation (equal); investigation (equal). **Francesca Vignolo Lutati:** Data curation (equal); investigation (equal). **Jacopo Azzolini:** Data curation (equal); investigation (equal). **Rita Danesi:** Data curation (equal); investigation (equal). **Valentina Arcangeli:** Data curation (equal); investigation (equal). **Sandrine M. Caputo:** Data curation (equal); investigation (equal). **Nadia Boutry‐Kryza:** Data curation (equal); investigation (equal). **Vincent Goussot:** Data curation (equal); investigation (equal). **Susan Hiraki:** Data curation (equal); investigation (equal). **Marcy Richardson:** Data curation (equal); investigation (equal). **Stefania Boni:** Data curation (equal). **Milena Gusella:** Data curation (equal). **Silvia Tognazzo:** Data curation (equal). **Isabella Mammi:** Data curation (equal). **Francesco Benedicenti:** Data curation (equal). **Licia Turolla:** Data curation (equal). **Fiorenza Soli:** Data curation (equal). **Simona Ferrari:** Data curation (equal); investigation (equal). **Paolo Radice:** Data curation (equal); investigation (equal). **Amanda B. Spurdle:** Data curation (equal); investigation (equal); methodology (equal); supervision (equal); writing – review and editing (equal). **Daniela Turchetti:** Conceptualization (equal); data curation (equal); investigation (equal); supervision (equal); writing – review and editing (equal).

## FUNDING INFORMATION

The work reported in this publication was funded by the Italian Ministry of Health, RC‐2023‐2,778,941/RC‐2024‐2,790,136. Further, ABS was supported by an NHMRC Investigator Fellowship (APP177524); the work of CF was supported by a grant from the National Breast Cancer Foundation, Australia (IIRS‐21‐102); the work of MTP was supported in part by National Institutes of Health grant U24 5U24CA258058–02; the work of LC and PR was supported by a grant from the Italian Association for Cancer Research (AIRC; IG22093).

## CONFLICT OF INTEREST STATEMENT

No authors have any conflict of interest to declare related with the work presented here.

## ETHIC STATEMENT

The study was conducted in accordance with the Declaration of Helsinki. All individuals had provided written informed consent to the use of their data for research purposes. The study was approved by the Ethical Board of “Area Vasta Emilia Centro” of Emilia‐Romagna region (CB‐AVEC), Italy (490/2022 /Oss/AOUBo).

## Supporting information


Figure S1:

Data S1:

## Data Availability

Individual patient data cannot be shared due to privacy or ethical restrictions. Requests for aggregate study data can be submitted to the corresponding author.
