# Correction to Atypical Cancer Risk Profile in Carriers of Italian Founder *BRCA1* Variant p.His1673del: Implications for Classification and Clinical Management

**DOI:** 10.1002/cam4.70464

**Published:** 2024-12-03

Innella G, Fortuno C, Caleca L, et al. Atypical cancer risk profile in carriers of Italian founder *BRCA1* variant p.His1673del: Implications for classification and clinical management. *Cancer Med*. 2024; 13:e70114. https://doi.org/10.1002/cam4.70114.

In the Affiliations section, affiliation number 2 was incorrect. This should read: Medical Genetics Unit, IRCCS Azienda Ospedaliero‐Universitaria di Bologna, Bologna, Italy.

We apologize for this error.

