# Supplementary figures and images for: Atypical cancer risk profile in carriers of Italian founder BRCA1 variant p.His1673del: Implications for classification and clinical management
Source: Cancer Med. 2024 Aug 28;13(16):e70114. doi: 10.1002/cam4.70114 (PMC11350839; doi:10.1002/cam4.70114)

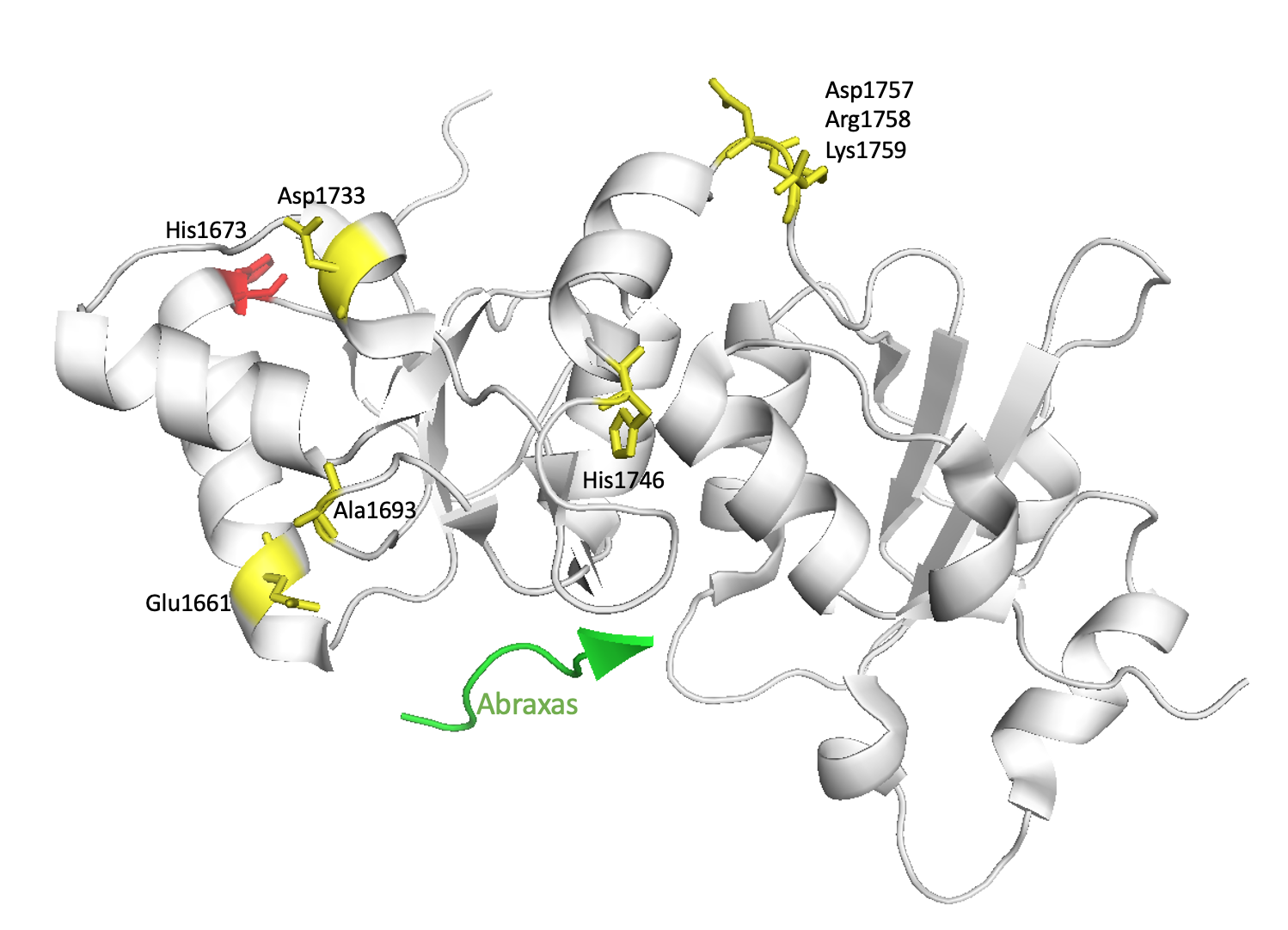

Supplement: Supplementary file 1 — Figure S1: [file CAM4-13-e70114-s001.png]
