# Supplementary material for: Atypical cancer risk profile in carriers of Italian founder BRCA1 variant p.His1673del: Implications for classification and clinical management
Source: Cancer Med. 2024 Aug 28;13(16):e70114. doi: 10.1002/cam4.70114 (PMC11350839; doi:10.1002/cam4.70114)
Supplement: Supplementary file 2 — Data S1: [file CAM4-13-e70114-s002.docx]

**Atypical cancer risk profile in carriers of Italian founder *BRCA1* variant p.His1673del: implications for classification and clinical management**

# **Supplementary Material**

# **Data collection**

# A database was set up including the following information: age at cancer diagnosis, if any; age at last follow-up or death; age at risk-reducing surgery for target organs (breast, ovary, uterus); histological type and grade of tumor (all cancers analyzed) and histopathological tumor data for BC, if available (estrogen receptor, progesterone receptor, and HER2 status). Individuals with multiple cancers were considered affected only for the tumor type that occurred first.

# Missing ages were handled as follows: for unaffected subjects, missing ages were imputed using other pedigree members through the PedPro program ([http://www.bjfenglab.org](http://www.bjfenglab.org/)); for affected patients with unknown age at cancer diagnosis, age at diagnosis was imputed as the first of age at pedigree construction, death, other cancer diagnosis, last follow-up or risk-reducing surgery - or - if there were no such age information, as the sex-specific average age at diagnosis of the corresponding cancer in the dataset, as previously performed[1].

# **Reassessment of breast and ovarian tumor LOH for predicting *BRCA1* (and *BRCA2*) variant pathogenicity**

The first study to apply *BRCA1* and *BRCA2* LOH for assessment of variant causality was Chenevix-Trench et al (2006)[2]. The hypothesis used to calculate likelihood estimates for breast tumor LOH was stated as follows: “To use the LOH data in our assessment of causality, we assumed that LOH for *BRCA1* and *BRCA2* occurs in 30% of sporadic tumors (PMID: 7577475, 7942844) compared with 80% of *BRCA1/BRCA2* tumors (PMID: 11979449, 7731724, 1303261, 10755399). Among *BRCA1/BRCA2* tumors that exhibit LOH, we assumed further that almost all (79 of 80; PMID: 11979449, 7669740, 7731724, 1303261, 7585515) lose the wild-type allele, whereas the allele loss is random in non-BRCA tumors with LOH. Thus, a tumor from an unclassified variant carrier that shows loss of the variant allele contributes odds of 15:1 against causality of this variant.” A second study from the same group[3] removed use of LOH from their classification process, justified as follows: “In this study, we did not include analysis of tumor loss of heterozygosity for *BRCA1* and *BRCA2*, as done previously (PMID: 16489001), because analysis of this and our previous data set revealed statistically significant increased loss of the variant compared with what is expected for the underlying hypothesis used previously to calculate likelihood estimates.” Annotation of LOH for these aforementioned studies was performed using microsatellite-based approaches.

More recently, a study by Santana Dos Santos et al (2022)[4] reported the utility of LOH analysis for *BRCA1* variant classification considering breast and ovarian tumors combined, using pyrosequencing technology to detect allelic imbalance for the majority of samples analyzed; *BRCA2* LOH was not assessed. Review of the data separated by tumor type revealed that LOH of the wildtype allele was observed for all of the limited number of ovarian cancer samples assessed (from four individuals with a pathogenic variant, and one with a benign variant). Given knowledge that there is frequent observation of *BRCA1* LOH in ovarian tumors overall, reported to be from 44% to 54% of tumors in previous studies[5-7], we concluded there was insufficient justification to apply *BRCA1* LOH in OC as a predictor of variant pathogenicity.

According to Santana Dos Santos et al (2022)[4], sequencing-derived BC LOH is a statistically significant predictor of *BRCA1* variant pathogenicity (Supplementary Table 1), however we elected to exclude LOH-based LR data for this study for several reasons: there was limited amount of information about LOH in BC in our dataset (available only for five cases); LRs derived for breast LOH are based on a much smaller sample set than that used to derive other breast tumor pathology features (grade, and ER, PR and HER status) used routinely in LR-based analyses[8]; lastly, LOH status correlated with these breast tumor features (Supplementary Table 2), and would thus not constitute an independent predictor of variant pathogenicity.

# **Assessment of BRCA1-ABRAXAS binding with *BRCA1* c.5017_5019del (p.His1673del) variant**

The majority of missense pathogenic variants mapped to the *BRCA1* and *BRCA2* genes occur in key conserved protein domains, including the RING-finger and BRCT domains of BRCA1 and the DNA binding domain (DBD) of BRCA2, all of which are involved in protein-protein interactions (PPIs)[9-12]. Thus, studying non-protein-truncating *BRCA* variants in the context of PPI networks can improve our understanding of their clinical relevance. Hence, in previous studies, gene region-specific functional assays were developed, based on the GFP-fragment reassembly technique, to study the effect on PPIs of patient-derived missense variants in *BRCA1* and *BRCA2*, and provide evidence in favour of or against pathogenicity of these variants[13, 14]. In this study, the same approach was applied to assess if the *BRCA1* c.5017_5019del (p.His1673del) affects the interaction of the BRCA1-BRCT domains with the binding protein ABRAXAS1.

# **Supplementary Table 1.** Re-analysis of pyrosequencing-derived breast tumor LOH data as a predictor of variant pathogenicity*

| **Source variant classification**  **(total number of breast tumors analyzed)** | **Loss of wildtype allele**  **N (%)** | **Loss of variant allele**  **N (%)** | **Allelic Balance**  **N (%)** |
| --- | --- | --- | --- |
| (Likely) Benign  (N=26) | 5 (19%) | 4 (15%) | 17 (65%) |
| Pathogenic  (N=51) | 32 (62%) | 3 (6%) | 16 (31%) |
| LR towards pathogenicity (95% CI) | 3.26 (1.44 to 7.38) | 0.40 (0.09 to 1.58) | 0.47 (0.29 to 0.79) |
| Evidence weight | Supporting Pathogenic | Supporting Benign | Supporting Benign |

**Data extracted from Santana Dos Santos et al (2022)[4] and analyzed through a Chi-square test (13.1446; p-value 0.001). ACMG/AMP evidence weight assigned following recommendations of Tavtigian et al (2018)[15].*

*LR = Likelihood Ratio; CI = Confidence Interval.*

# **Supplementary Table 2.** Re-analysis of pyrosequencing-derived breast tumor LOH data to assess correlation with tumor histopathological predictors of variant pathogenicity*.

| **LOH result**  **(total number of breast tumors analyzed)** | **High grade**  **N (%)** | **Other or unknown grade**  **N (%)** | **Correlation** | **Triple-negative or ER-negative, PR- negative, HER2 unknown** | **Any marker positive or unknown status** | **Correlation** |
| --- | --- | --- | --- | --- | --- | --- |
| Loss of Wildtype allele  (37) | 34 (91.9%) | 3 (8.1%) | Chi-square = 13.8196.  *p*-value = 0.000998. | 27 (73.0%) | 10 (27.0%) | Chi-square = 12.6655.  *p*-value = 0.001777. |
| Loss of Variant allele  (7) | 3 (42.9%) | 4 (57.1%) |  | 2 (28.6%) | 5 (71.4%) |  |
| Allelic Balance  (33) | 19 (67.6%) | 14 (32.4%) |  | 11 (33.3%) | 22 (66.7%) |  |

**Data extracted from Santana Dos Santos et al (2022)[4] and analyzed through a Chi-square test. Data for pathogenic and benign variant carriers collapsed to assess correlation.*

*ER = Estrogen-receptor breast tumor status; PR = Progesterone-receptor breast tumor status; HER2 = HER2 breast tumor status.*

# **Supplementary Table 3.** Main features of families included in the study divided by sex.

| **Females** | | | | | | | | | | | | | | | | | | | |
| --- | --- | --- | --- | --- | --- | --- | --- | --- | --- | --- | --- | --- | --- | --- | --- | --- | --- | --- | --- |
| **Age**  **(years)** | **Carriers** | | | | | | **Noncarriers** | | | | | | **Untested** | | | | | | **Total** |
|  | **Tot.** | **BC** | **OC** | **PC** | **CRC** | **UC** | **Tot.** | **BC** | **OC** | **PC** | **CRC** | **UC** | **Tot.** | **BC** | **OC** | **PC** | **CRC** | **UC** |  |
| **≤30** | 4 | 0 | 0 | 0 | 0 | 0 | 1 | 0 | 0 | 0 | 0 | 0 | 4 | 0 | 0 | 0 | 0 | 0 | 9 |
| **31-40** | 21 | 8 | 3 | 0 | 1 | 0 | 10 | 2 | 0 | 0 | 0 | 0 | 22 | 8 | 1 | 0 | 1 | 2 | 53 |
| **41-50** | 58 | 19 | 25 | 0 | 0 | 3 | 15 | 4 | 0 | 0 | 0 | 0 | 28 | 8 | 6 | 0 | 0 | 0 | 101 |
| **51-60** | 27 | 5 | 16 | 0 | 0 | 1 | 12 | 3 | 0 | 0 | 1 | 0 | 42 | 12 | 10 | 1 | 1 | 1 | 81 |
| **61-70** | 20 | 6 | 14 | 0 | 1 | 0 | 4 | 0 | 0 | 0 | 0 | 0 | 21 | 3 | 4 | 1 | 3 | 1 | 45 |
| **71-80** | 7 | 1 | 6 | 0 | 0 | 0 | 4 | 1 | 0 | 0 | 0 | 0 | 20 | 1 | 2 | 0 | 1 | 0 | 31 |
| **>80** | 3 | 0 | 1 | 0 | 0 | 0 | 0 | 0 | 0 | 0 | 0 | 0 | 36 | 2 | 0 | 0 | 0 | 0 | 39 |
| **Unknown** | 10 | 0 | 0 | 0 | 0 | 0 | 4 | 0 | 0 | 0 | 0 | 0 | 109 | 3 | 2 | 0 | 1 | 1 | 123 |
| **Total** | 150 | 39 | 65 | 0 | 2 | 4 | 50 | 10 | 0 | 0 | 1 | 0 | 282 | 37 | 25 | 2 | 6 | 5 | 482 |

| **Males** | | | | | | | | | | | | | | | | |
| --- | --- | --- | --- | --- | --- | --- | --- | --- | --- | --- | --- | --- | --- | --- | --- | --- |
| **Age**  **(years)** | **Carriers** | | | | | **Noncarriers** | | | | | **Untested** | | | | | **Total** |
|  | **Tot.** | **BC** | **ProC** | **PC** | **CRC** | **Tot.** | **BC** | **ProC** | **PC** | **CRC** | **Tot.** | **BC** | **ProC** | **PC** | **CRC** |  |
| **≤30** | 3 | 0 | 0 | 0 | 0 | 2 | 0 | 0 | 0 | 0 | 2 | 0 | 0 | 0 | 0 | 7 |
| **31-40** | 3 | 0 | 0 | 0 | 0 | 3 | 0 | 0 | 0 | 0 | 1 | 0 | 0 | 0 | 0 | 7 |
| **41-50** | 7 | 0 | 0 | 0 | 0 | 4 | 0 | 0 | 0 | 0 | 6 | 0 | 0 | 0 | 0 | 17 |
| **51-60** | 2 | 0 | 0 | 0 | 0 | 0 | 0 | 0 | 0 | 0 | 10 | 0 | 1 | 0 | 1 | 12 |
| **61-70** | 1 | 0 | 0 | 0 | 1 | 1 | 0 | 0 | 0 | 0 | 16 | 0 | 1 | 0 | 1 | 18 |
| **71-80** | 6 | 0 | 0 | 0 | 0 | 0 | 0 | 0 | 0 | 0 | 14 | 0 | 0 | 0 | 0 | 20 |
| **>80** | 2 | 0 | 1 | 0 | 0 | 1 | 0 | 0 | 0 | 0 | 12 | 0 | 0 | 0 | 0 | 15 |
| **Unknown** | 6 | 0 | 0 | 0 | 0 | 2 | 0 | 0 | 0 | 0 | 205 | 0 | 0 | 0 | 1 | 213 |
| **Total** | 30 | 0 | 1 | 0 | 1 | 13 | 0 | 0 | 0 | 0 | 266 | 0 | 2 | 0 | 3 | 309 |

*BC = Breast Cancer; OC = Ovarian Cancer; PC = Pancreatic Cancer; CRC = ColoRectal Cancer; ProC = Prostate Cancer; UC = Uterine Cancer.*

# **Supplementary Table 4.** Clinical-pathological features of breast and ovarian cancer occurring in Italian carriers of c.5017_5019del (p.His1673del) variant.

| **Feature** | | **BC** | **OC** |
| --- | --- | --- | --- |
| **Number of cases (N)** | | 39 | 65 |
| **Average age at diagnosis (years)** | | 50 | 56 |
| **Stage^§^**  **N/Total* (%)** | **I** | 13/26  (50.0%) | 5/40  (12.5%) |
|  | **II** | 11/26  (42.3%) | 7/40  (17.5%) |
|  | **III** | 2/26  (7.7%) | 23/40  (57.5%) |
|  | **IV** | 0/26  (0.0%) | 3/40  (7.5%) |
| **Grade**  **N/Total* (%)** | **1** | 1/27  (3.7%) | 0/48  (0.0%) |
|  | **2** | 11/27  (40.7%) | 1/48  (2.1%) |
|  | **3** | 15/27  (55.6%) | 47/48  (97.9%) |
| **Histological type**  **N/Total* (%)** | **BC: Ductal** | 26/31  (83.9%) | / |
|  | **BC: Lobular** | 3/31  (9.7%) | / |
|  | **BC: Unknown/Other** | 2/31  (6.5%) | / |
|  | **OC: Serous** | / | 41/43  (95.3%) |
|  | **OC: Endometrioid** | / | 2/43  (4.7%) |
| **BC receptor status**  **N/Total* (%)** | **Hormone-responsive^#^** | 19/29  (65.5%) | / |
|  | **Triple-negative^#^** | 10/28  (35.7%) | / |

** of patients for whom the information was available.*

*^§^ AJCC 2009 for BC. International Federation of Gynecology and Obstetrics (FIGO) for OC.*

*^#^ considered hormone responsive if at least one of the estrogen and progesterone receptors was expressed, triple negative if neither hormone receptors nor HER2 were expressed.*

*BC = Breast Cancer; OC = Ovarian Cancer.*

# **Supplementary Table 5.** HRs and cumulative risks for breast and ovarian cancer associated with c.5017_5019del (p.His1673del) variant, considering different age group cut-offs.

| **BREAST CANCER** | | | | | |
| --- | --- | --- | --- | --- | --- |
| **Age cut-offs** | **<50/≥50 years** | **≤40/40-60/≥60 years*** | **≤30/30-50/≥50 years*** | **≤40/40-50/≥50 years*** | **≤50/50-60/≥60 years*** |
| **HR (95% CI)** | <50: 1.3 (0.4-4.5)  ≥50: 0.4 (0.1-2.0) | ≤40: 1.2 (0.3-4.3)  ≥60: 0.3 (0.0-3.3) | ≤30: 1.5 (0.1-23.8)  ≥50: 0.6 (0.1-2.9) | ≤40: 1.1 (0.2-7.8)  ≥50: 0.6 (0.1-2.9) | ≤50: 1.0 (0.3-3.0)  ≥60: 0.3 (0.0-3.0) |
| **Age (years)** | **% Cumulative risk (95% CI)** | | | | |
| **25** | 0.1 (0.0-0.2) | 0.1 (0.0-0.2) | 0.2 (0.0-1.2) | 0.1 (0.0-0.4) | 0.1 (0.0-0.1) |
| **30** | 0.3 (0.1-0.8) | 0.3 (0.1-0.8) | 0.7 (0.0-4.3) | 0.3 (0.0-1.4) | 0.2 (0.1-0.5) |
| **35** | 0.8 (0.2-2.4) | 0.8 (0.2-2.2) | 2.0 (0.0-11.9) | 1.0 (0.0.1-4) | 0.6 (0.2-1.5) |
| **40** | 1.9 (0.4-5.5) | 1.8 (0.4-5.1) | 4.5 (0.1-25.9) | 2.3 (0.2-9.3) | 1.4 (0.4-3.5) |
| **45** | 3.2 (0.8-9.1) | 3.1 (0.8-8.5) | 7.1 (0.5-39.1) | 3.9 (0.6-15.0) | 2.4 (0.7-6.0) |
| **50** | 4.4 (1.2-11.9) | 4.3 (1.2-11.3) | 9.2 (1.0-47.5) | 5.3 (1.1-19.1) | 3.5 (1.1-8.5) |
| **55** | 5.4 (1.6-13.9) | 5.4 (1.6-13.9) | 10.9 (1.6-51.7) | 6.6 (1.6-21.7) | 4.4 (1.3-11.0) |
| **60** | 6.2 (1.9-15.2) | 6.3 (1.8-16.6) | 12.0 (2.2-51.9) | 7.8 (2.1-23.3) | 5.3 (1.5-14.4) |
| **65** | 7.1 (2.1-17.1) | 7.3 (2.1-20.0) | 13.2 (2.8-52.2) | 9.0 (2.5-25.0) | 6.2 (1.7-17.8) |
| **70** | 7.8 (2.3-19.0) | 8.2 (2.3-23.4) | 14.3 (3.2-52.5) | 10.0 (2.8-26.4) | 7.0 (1.8-21.1) |

| **OVARIAN CANCER** | | | | |
| --- | --- | --- | --- | --- |
| **Age cut-offs** | **<50/≥50 years** | **≤40/40-60/≥60 years*** | **≤50/50-60/≥60 years*** | **<60//≥60 years** |
| **HR (95% CI)** | <50: 31.1 (3.3-292.4)  ≥50: 71.6 (7.4-692.8) | ≤40: 12.5 (0.1-1730.8)  ≥60: 95.1 (13.1-692.6) | ≤50: 36.3 (4.4-301.5)  ≥60: 71.3 (4.0-1256.9) | <60: 38.1 (5.4-271.0)  ≥60: 76.4 (2.7-2198.3) |
| **Age (years)** | **% Cumulative risk (95% CI)** | | | |
| **25** | 1.4 (0.1-6.8) | 3.2 (0.0-29.0) | 1.6 (0.1-7.5) | 1.5 (0.1-6.2) |
| **30** | 2.3 (0.1-11.1) | 4.7 (0.0-43.5) | 2.6 (0.2-12.2) | 2.4 (0.2-10.0) |
| **35** | 3.8 (0.2-18.3) | 6.8 (0.0-62.4) | 4.3 (0.3-20.0) | 4.0 (0.4-16.6) |
| **40** | 6.2 (0.4-28.9) | 9.6 (0.0-80.8) | 6.9 (0.5-31.4) | 6.6 (0.6-26.3) |
| **45** | 11.7 (1.5-43.4) | 15.5 (0.9-92.4) | 13.8 (1.9-51.0) | 14.7 (2.1-49.4) |
| **50** | 19.8 (3.2-62.8) | 24.3 (2.6-96.7) | 23.3 (3.6-74.3) | 25.1 (3.8-82.9) |
| **55** | 28.9 (5.2-80.5) | 34.7 (5.3-98.5) | 33.2 (5.4-90.9) | 35.2 (5.4-97.8) |
| **60** | 38.0 (7.3-92.0) | 45.2 (8.5-99.3) | 42.1 (7.0-98.4) | 43.7 (6.9-99.9) |
| **65** | 45.6 (9.1-97.6) | 53.9 (12.0-99.8) | 49.1 (8.6-99.8) | 50.3 (8.0-100.0) |
| **70** | 51.9 (11.1-99.4) | 60.7 (15.5-99.9) | 54.7 (10.1-100.0) | 55.4 (8.8-100.0) |

**For models with three age-groups the HR for the intermediate age group is interpolated.*

*HR = Hazard Ratio; CI = Confidence Interval.*

# **Supplementary Table 6.** HRs and cumulative risks for ovarian cancer associated with c.5017_5019del (p.His1673del) variant, calculated using population incidence data related to all ovarian cancers (Main analysis) and population incidence data related to only epithelial ovarian cancers added to that of tubal cancers (Secondary analysis).

|  | **Main Analysis** | **Secondary analysis** |
| --- | --- | --- |
| **HR (95% CI)** | 45.5 (8.8-236.1) | 53.4 (9.2-310.3) |
| **Age (years)** | **% Cumulative risk (95% CI)** | |
| **25** | 1.3 (0.3-2.3) | 0.5 (0.0-1.2) |
| **30** | 2.0 (0.4-3.6) | 1.1 (0.0-2.4) |
| **35** | 3.4 (0.6-6.0) | 2.4 (0.0-4.9) |
| **40** | 5.5 (1.0-9.8) | 4.6 (0.0-9.1) |
| **45** | 9.3 (1.6-16.4) | 8.3 (0.2-15.8) |
| **50** | 14.1 (3.0-24.0) | 13.2 (1.3-23.6) |
| **55** | 19.7 (5.4-31.9) | 18.8 (3.4-31.8) |
| **60** | 25.7 (8.5-39.6) | 24.7 (6.3-39.5) |
| **65** | 32.0 (12.3-47.3) | 30.9 (9.9-47.1) |
| **70** | 38.2 (16.7-54.2) | 36.6 (13.9-53.3) |

*HR = Hazard Ratio; CI = Confidence Interval.*

# **Supplementary Table 7.** Co-segregation analysis results with standard *BRCA1* penetrance assumptions (Main analysis), and considering individuals affected by breast cancer as unaffected at age at breast cancer diagnosis (Secondary analysis).

| **Pedigree** | **Main analysis** | | **Secondary analysis** | |
| --- | --- | --- | --- | --- |
|  | **LOD-score** | **LR** | **LOD-score** | **LR** |
| 2 | 1.62104 | 41.78689 | 0.95802 | 9.078623378 |
| 3 | -0.48344 | 0.328522 | 0.127009 | 1.33970445 |
| 4 | 0.246292 | 1.763161 | 0.246292 | 1.763161118 |
| 5 | 0.290504 | 1.952109 | -0.0280389 | 0.937478033 |
| 6 | -0.15598 | 0.698258 | 0.190905 | 1.552047469 |
| 7 | -1.1152 | 0.076701 | -1.06441 | 0.086216423 |
| 8 | -0.46061 | 0.346249 | -0.217898 | 0.605483064 |
| 11 | -0.08707 | 0.818342 | -0.087065 | 0.818342299 |
| 12 | 0.157731 | 1.437908 | 0.1856 | 1.533204199 |
| 13 | 0.457524 | 2.867636 | 0.457524 | 2.867635843 |
| 14 | 0.039945 | 1.096338 | 0.0399446 | 1.096338335 |
| 15 | -0.26259 | 0.546268 | -0.262594 | 0.546268301 |
| 17 | 0.195992 | 1.570334 | 0.195992 | 1.570333877 |
| 19 | -0.00458 | 0.989518 | -0.00457645 | 0.989517661 |
| 20 | -0.6024 | 0.249805 | -0.578302 | 0.264057192 |
| 21 | 0.072508 | 1.181701 | 0.0725075 | 1.18170072 |
| 23 | -0.40912 | 0.389839 | -0.409115 | 0.389838745 |
| 24 | -0.50882 | 0.309872 | 0.033824 | 1.080995784 |
| 25 | 0.000251 | 1.000577 | 0.000269327 | 1.000620341 |
| 26 | 0.291116 | 1.954862 | 0.291116 | 1.954861529 |
| 27 | 0.072508 | 1.181701 | 0.0725075 | 1.18170072 |
| 28 | -0.08707 | 0.818336 | -0.0870685 | 0.818335704 |
| 30 | 0.144456 | 1.39462 | 0.144456 | 1.394620356 |
| 31 | 0.193958 | 1.562996 | 0.193958 | 1.56299648 |
| 32 | -0.02801 | 0.937539 | -0.0280107 | 0.937538908 |
| 33 | -0.00324 | 0.992568 | -0.00323982 | 0.992567795 |
| 34 | -0.41096 | 0.388183 | 0.19948 | 1.582996665 |
| 35 | -0.00147 | 0.996629 | 0.0470469 | 1.114414874 |
| 36 | 0.026318 | 1.062473 | 0.0263181 | 1.062473483 |
| 37 | 0.121245 | 1.322041 | 0.121245 | 1.322041231 |
| 38 | 1.60788 | 40.53965 | 1.98776 | 97.22098123 |
| 39 | -0.08707 | 0.818336 | -0.0870685 | 0.818335704 |
| 40 | 0.398708 | 2.504425 | 0.398708 | 2.504424825 |
| 41 | 0.578456 | 3.788402 | 0.224592 | 1.677227598 |
| 44 | 0.355876 | 2.269217 | -0.064103 | 0.862773903 |
| 46 | -0.02723 | 0.93922 | -0.0271711 | 0.939353159 |
| 50 | -0.0065 | 0.985135 | -0.00650436 | 0.985134753 |
| 52 | 0.003221 | 1.007443 | 0.00322055 | 1.007443154 |
| 53 | -2.90E-05 | 0.999933 | -2.90E-05 | 0.999933254 |
| 56 | 0.11334 | 1.298195 | -0.517083 | 0.304030392 |
| 57 | -0.80232 | 0.157644 | 0.0900667 | 1.230457733 |
| 59 | -0.02801 | 0.937539 | -0.0280107 | 0.937538908 |
| 61 | -0.18015 | 0.660473 | -0.180145 | 0.660472896 |
| 62 | 0.129038 | 1.345978 | -0.36983 | 0.426746532 |
| 63 | 0.564615 | 3.669569 | 0.564634 | 3.669729051 |
| 64 | 3.82E-06 | 1.000009 | 3.82E-06 | 1.000008794 |
| 65 | 0.180327 | 1.514701 | 0.180327 | 1.514701306 |
| 67 | -0.21142 | 0.614586 | -0.211417 | 0.614586477 |
| 68 | 0.126989 | 1.339643 | 0.126998 | 1.339670518 |
| 69 | 1.04823 | 11.17455 | 1.04823 | 11.17454889 |
| 70 | -0.81982 | 0.151418 | 0.0725224 | 0.151417824 |
| 71 | -0.48351 | 0.328468 | 0.126995 | 1.339661264 |
| 72 | 0.28776 | 1.939814 | 0.287799 | 1.939987805 |
| **Tot.** | 2.059221481 | **114.6097278** | 4.454191378 | **3646.246103** |

*LR = Likelihood Ratio.*

# **Supplementary Table 8.** Application of pathology likelihood ratios to breast cancer cases occurring in Italian carriers of c.5017_5019del (p.His1673del) variant.

| **Individual** | **Age at diagnosis (years)** | **Grade** | **ER** | **PR** | **HER2** | **LR** |
| --- | --- | --- | --- | --- | --- | --- |
| 2-8 | 51 | 2 | Pos | Pos | Neg | 0.34 |
| 5-2 | 38 | Unk | Pos | Neg | Neg | 0.4 |
| 7-1 | 56 | 3 | Neg | Neg | Neg | 4.13 |
| 7-3 | 48 | 2 | Pos | Pos | Neg | 0.21 |
| 10-1 | 69 | 2 | Pos | Pos | Neg | 0.34 |
| 11-1 | 32 | 3 | Pos | Pos | Pos | 0.64 |
| 12-1 | 72 | 1 | Pos | Pos | Neg | 0.11 |
| 14-1 | 65 | 3 | Neg | Neg | Neg | 4.13 |
| 16-1 | 43 | 3 | Neg | Neg | Neg | 3.16 |
| 23-1 | 47 | 2 | Pos | Pos | Neg | 0.21 |
| 26-1 | 47 | 3 | Pos | Neg | Neg | 0.64 |
| 29-1 | 53 | 3 | Neg | Neg | Neg | 4.13 |
| 32-1 | 42 | Unk | Pos | Pos | Unk | 0.32 |
| 35-1 | 48 | 2 | Pos | Pos | Neg | 0.21 |
| 36-1 | 38 | 3 | Neg | Neg | Neg | 3.16 |
| 38-1 | 38 | 3 | Pos | Pos | Neg | 0.64 |
| 40-2 | 57 | 2 | Pos | Pos | Pos | 0.34 |
| 41-3 | 42 | Unk | Neg | Pos | Neg | 0.4 |
| 42-1 | 46 | 2 | Pos | Pos | Neg | 0.21 |
| 43-1 | 49 | 2 | Unk | Unk | Unk | 0.38 |
| 44-1 | 49 | 2 | Pos | Pos | Neg | 0.21 |
| 46-1 | 47 | 3 | Pos | Pos | Neg | 0.64 |
| 52-1 | 57 | 2 | Pos | Pos | Neg | 0.34 |
| 53-1 | 39 | 3 | Neg | Neg | Neg | 3.16 |
| 54-1 | 39 | 3 | Neg | Neg | Neg | 3.16 |
| 56-2 | 86 | 3 | Unk | Unk | Unk | 2.2 |
| 61-1 | 70 | 2 | Pos | Pos | Neg | 0.34 |
| 62-1 | 47 | 3 | Neg | Neg | Neg | 3.16 |
| 65-1 | 50 | 3 | Neg | Neg | Neg | 4.13 |
| 67-1 | 54 | 3 | Pos | Neg | Pos | 0.9 |
| 72-1 | 46 | 3 | Neg | Neg | Neg | 3.16 |
| **Total** |  | | | | | **0.00038** |

*ER = Estrogen-receptor breast tumor status; PR = Progesterone-receptor breast tumor status; HER2 = HER2 breast tumor status; LR = Likelihood Ratio; Pos = Positive; Neg = Negative; Unk = Unknown.*

# **Supplementary Table 9.** Application of pathology likelihood ratios to ovarian cancer cases occurring in Italian carriers of c.5017_5019del (p.His1673del) variant.

| **Histological type** | **Number of cases** | **LR** |
| --- | --- | --- |
| HGSC | 41 | Not informative |
| Endometrioid | 2 | 8.8804 |
| **Total** |  | **8.8804** |

*LR = Likelihood Ratio; HGSC = High-Grade Serous Carcinoma.*

# **Supplementary Figure 1.** Protein modelling of codons involved in small in-frame indels tested in this work.

*Modelling used the 4Y2G structure of BRCT repeats of BRCA1 in interaction with Abraxas[16]. Model of the selected codons was visualized with PYMOL software (DeLano Scientific LLC, Palo Alto, CA). Abraxas is shown in green, the 1673 codon in red and other codons in yellow.*

# **REFERENCES**

1. Li, S., Silvestri, V., Leslie, G., Rebbeck, T. R., Neuhausen, S. L., Hopper, J. L., et al. (2022). Cancer Risks Associated With BRCA1and BRCA2 Pathogenic Variants. Journal of clinical oncology : official journal of the American Society of Clinical Oncology, 40(14), 1529–1541. https://doi.org/10.1200/JCO.21.02112
2. Chenevix-Trench, G., Healey, S., Lakhani, S., Waring, P., Cummings, M., Brinkworth, R., et al. (2006). Genetic and histopathologic evaluation of BRCA1 and BRCA2 DNA sequence variants of unknown clinical significance. Cancer research, 66(4), 2019–2027. https://doi.org/10.1158/0008-5472.CAN-05-3546
3. Spurdle, A. B., Lakhani, S. R., Healey, S., Parry, S., Da Silva, L. M., Brinkworth, R., et al. (2008). Clinical classification of BRCA1 and BRCA2 DNA sequence variants: the value of cytokeratin profiles and evolutionary analysis--a report from the kConFab Investigators. Journal of clinical oncology: official journal of the American Society of Clinical Oncology, 26(10), 1657–1663. https://doi.org/10.1200/JCO.2007.13.2779
4. Santana Dos Santos, E., Spurdle, A. B., Carraro, D. M., Briaux A., Southey M., Torrezan G., et al (2022). Value of the loss of heterozygosity to BRCA1 variant classification. NPJ breast cancer, 8(1), 9. <https://doi.org/10.1038/s41523-021-00361-2>
5. Russell, P. A., Pharoah, P. D., De Foy, K., Ramus, S. J., Symmonds, I., Wilson, A., et al. (2000). Frequent loss of BRCA1 mRNA and protein expression in sporadic ovarian cancers. International journal of cancer, 87(3), 317–321. https://doi.org/10.1002/1097-0215(20000801)87:3<317::aid-ijc2>3.0.co;2-b
6. Geisler, J. P., Hatterman-Zogg, M. A., Rathe, J. A., & Buller, R. E. (2002). Frequency of BRCA1 dysfunction in ovarian cancer. Journal of the National Cancer Institute, 94(1), 61–67. https://doi.org/10.1093/jnci/94.1.61
7. Brozek, I., Ochman, K., Debniak, J., Morzuch, L., Ratajska, M., Stepnowska, M., et al. (2009). Loss of heterozygosity at BRCA1/2 loci in hereditary and sporadic ovarian cancers. Journal of applied genetics, 50(4), 379–384. https://doi.org/10.1007/BF03195697
8. Spurdle, A. B., Couch, F. J., Parsons, M. T., McGuffog, L., Barrowdale, D., Bolla, M. K., et al. (2014). Refined histopathological predictors of BRCA1 and BRCA2 mutation status: a large-scale analysis of breast cancer characteristics from the BCAC, CIMBA, and ENIGMA consortia. Breast cancer research : BCR, 16(6), 3419. https://doi.org/10.1186/s13058-014-0474-y
9. Gudmundsdottir K, Ashworth A. The roles of BRCA1 and BRCA2 and associated proteins in the maintenance of genomic stability. Oncogene. 2006 Sep 25;25(43):5864-74. doi: 10.1038/sj.onc.1209874. PMID: 16998501.
10. Easton DF, Deffenbaugh AM, Pruss D, Frye C, Wenstrup RJ, Allen-Brady K, Taet al. A systematic genetic assessment of 1,433 sequence variants of unknown clinical significance in the BRCA1 and BRCA2 breast cancer-predisposition genes. Am J Hum Genet. 2007 Nov;81(5):873-83. doi: 10.1086/521032. Epub 2007 Sep 6. PMID: 17924331; PMCID: PMC2265654.
11. Sweet K, Senter L, Pilarski R, Wei L, Toland AE. Characterization of BRCA1 ring finger variants of uncertain significance. Breast Cancer Res Treat. 2010 Feb;119(3):737-43. doi: 10.1007/s10549-009-0438-6. Epub 2009 Jun 20. PMID: 19543972; PMCID: PMC4283813.
12. Venkitaraman AR. Cancer suppression by the chromosome custodians, BRCA1 and BRCA2. Science. 2014 Mar 28;343(6178):1470-5. doi: 10.1126/science.1252230. PMID: 24675954.
13. Caleca, L., Colombo, M., van Overeem Hansen, T., Lázaro, C., Manoukian, S., Parsons, M. T., et al. (2019). GFP-Fragment Reassembly Screens for the Functional Characterization of Variants of Uncertain Significance in Protein Interaction Domains of the BRCA1 and BRCA2 Genes. Cancers, 11(2), 151. https://doi.org/10.3390/cancers11020151
14. Caleca L, Radice P. Refinement of the assignment to the ACMG/AMP BS3 and PS3 criteria of eight BRCA1 variants of uncertain significance by integrating available functional data with protein interaction assays. Front Oncol. 2023 Apr 24;13:1146604. doi: 10.3389/fonc.2023.1146604. PMID: 37168384; PMCID: PMC10164951.
15. Tavtigian, S. V., Greenblatt, M. S., Harrison, S. M., Nussbaum, R. L., Prabhu, S. A., Boucher, K. M., et al. (2018). Modeling the ACMG/AMP variant classification guidelines as a Bayesian classification framework. Genetics in medicine : official journal of the American College of Medical Genetics, 20(9), 1054–1060. <https://doi.org/10.1038/gim.2017.210>
16. Wu, Q., Paul, A., Su, D., Mehmood, S., Foo, T. K., Ochi, T., et al. (2016). Structure of BRCA1-BRCT/Abraxas Complex Reveals Phosphorylation-Dependent BRCT Dimerization at DNA Damage Sites. Molecular cell, 61(3), 434–448. https://doi.org/10.1016/j.molcel.2015.12.017
